# Combinations of bacterial cultures, exogenous enzymes, and yeast-based feed additives and their impact on ruminal microbiome

**DOI:** 10.1093/tas/txac157

**Published:** 2022-12-06

**Authors:** J A Arce-Cordero, S L Bennett, T Liu, A Ravelo, R R Lobo, K C Jeong, A P Faciola

**Affiliations:** Department of Animal Sciences, University of Florida, Gainesville, FL 32611; Escuela de Zootecnia, Universidad de Costa Rica, San Jose, 11501-2060, Costa Rica; Department of Animal Sciences, University of Florida, Gainesville, FL 32611; Emerging Pathogens Institute, University of Florida, Gainesville, FL 32611; Department of Animal Sciences, University of Florida, Gainesville, FL 32611; Department of Animal Sciences, University of Florida, Gainesville, FL 32611; Emerging Pathogens Institute, University of Florida, Gainesville, FL 32611; Department of Animal Sciences, University of Florida, Gainesville, FL 32611

**Keywords:** butyrate, direct fed microbials, in vitro, propionate

## Abstract

Our objective was to evaluate the effects of bacteria (*Lactobacillus animalis, Propionibacterium freudenreichii, Bacillus lichenformis, Bacillus subtilis, and Enterococcus faecium*), enzymes (amylase, hemicellulose, and xylanase), and yeast as additives on the ruminal microbiome. We hypothesized that inclusion of bacteria, enzymes, and yeast would impact butyric bacterial populations. Eight fermenters were arranged in a duplicated 4 × 4 Latin square with the following treatments: 1) control without additives (CTRL); 2) bacterial culture and enzyme blend (EB); 3) bacterial culture and enzyme blend with a live yeast and yeast culture blend (EBY); and 4) double dose of bacterial culture and enzyme blend and the yeast products blend (2X). We conducted four fermentation periods of 10 d each, with the last 3 d for collection of samples. Overall, 64 solid and liquid samples were analyzed by amplification of the V4 region of bacterial 16S rRNA. Data were analyzed with R and SAS. The following orthogonal contrasts were used: 1) ADD—the control compared to all treatments with additives (CTRL vs. EB, EBY, and 2X); 2) YEAST—treatment without yeast compared to those with yeast (EB vs. EBY and 2X); and 3) DOSE—the single dose of enzymes, bacteria, and yeast compared to the doubled dose (EBY vs. 2X). Family *Prevotellaceae* was more abundant when additives were added (ADD). Additives (ADD) also increased relative abundance of *Prevotellaceae Ga6A1* and *YAB2003* in solid fraction, and of *Prevotellaceae Ga6A1* and two members of *Lachnospiracea* family in liquid fraction. Yeast (YEAST) decreased relative abundance of *Succinivibrionaceae UCG-001* and increased abundance of *Ruminococcus* and *Prevotellaceae UCG-003* in solid fraction. Doubling the dose of enzymes and microbial additives (DOSE) decreased the abundance of *Succiniclasticum* in solid fraction and *Selenomonadaceae* in the liquid. Molar proportion of butyrate was highly correlated with abundance of *Prevotellaceae Ga6A1* in solid (*r* = 0.68) and liquid fraction (*r* = 0.79), and with *Unclassified Lachnospiraceae* in liquid (*r* = 0.70). Our results demonstrate that YEAST decreases abundance of succinate synthesizing bacteria, while DOSE decreases abundance of bacteria that metabolize succinate into propionate. Combined bacteria, enzymes, and yeast increase the relative abundance of specific genera primarily within the *Prevotellaceae* family, which may explain the increase in butyrate molar proportion observed with ADD.

## INTRODUCTION

Previous research has evaluated the individual effects of bacterial cultures, enzymes, and yeasts on ruminal fermentation and animal productivity (Beauchemin et al., 2003; Desnoyers et al., 2009; Krehbiel et al., 2003). The use of lactate-producing and lactate-utilizing bacteria (Nocek et al., 2002) and propionic bacteria (Krehbiel et al., 2003) is supported by their role in stabilization of ruminal pH and increase in ruminal concentration of propionate, respectively. Yeast based direct-fed microbials can promote the growth of bacteria within the rumen through the promotion of an anaerobic environment (Newbold et al., 1996), which enhances conditions for lactate-utilizing bacteria and fibrolytic bacteria (Yoon and Stern, 1995). The supplementation of yeast also can prevent lactate accumulation and increase VFA synthesis in the rumen (Chaucheyras-Durand et al., 2008), further changing ruminal conditions. The supplementation of exogenous enzymes, such as xylanase, hemicellulase, and amylase can improve fermentation and digestion of feed (Arriola et al., 2017; Beauchemin et al., 2003; Meale et al., 2014), yielding more substrate available to ruminal microorganisms. Exogenous enzymes added to the diet have been shown to shift ruminal microbial communities (Chung et al., 2012).

Although the effects of these additives have been evaluated individually, diets fed in dairy cattle operations in the United States commonly include a combination of feed additives, including bacterial cultures, enzymes, and yeast. These have been tested in experiments comparing the effects of direct-fed microbials versus enzymes (Oh et al., 2019), and the combination of both yeast and bacterial cultures (Nocek and Kautz, 2006; Nocek et al., 2002); but to our knowledge not all forms of additives together. In our companion study (Bennett et al., 2021) we found that inclusion of additives (bacterial cultures, enzymes, and yeast) in a corn silage-based diet, increased the molar proportion of butyrate and tended to decrease propionate in ruminal fluid. To our knowledge, the potential additive effect of combined supplementation of microbial additives and enzymes on ruminal microbial populations has not been evaluated yet, which represents a gap in knowledge and an opportunity to better understand animal response when such additives are included in diets for dairy cows.

Our objective in the present study was to evaluate the possible effects of combinations of bacteria, enzymes, and yeast as additives in corn silage-based diets on ruminal microbiome. Based on the results of our companion study (Bennett et al., 2021) we hypothesized that inclusion of bacterial cultures, enzymes, and yeast would have an impact on bacterial populations associated with synthesis of butyrate.

## MATERIALS AND METHODS

All the procedures for care and handling of animals required for this experiment were in accordance with the Guide for the Care and Use of Agricultural Animals in Research and Teaching (FASS, 2010) and conducted under protocols that were approved by Institutional Animal Care and Use Committee at the University of Florida.

### Experimental Design and Diets

This is a companion study to Bennett et al. (2021). Eight fermenters of a dual-flow continuous culture system were arranged in a duplicated 4 × 4 Latin square design with treatments defined by combinations of additives that were added to a common basal diet (Table 1). Treatments were: 1) control without additives (CTRL); 2) bacterial culture and enzyme blend (EB); 3) bacterial culture and enzyme blend with a live yeast and yeast culture blend (EBY); and 4) double dose of bacterial culture and enzyme blend and the yeast products blend (2X). The basal diet used for all treatments was formulated to meet the NRC (2001) recommendations for a high producing lactating Holstein cow with 680 kg body weight and daily milk production of 45 kg.

**Table 1.** Ingredient and chemical composition of experimental diets

Item	Treatment^*a*^			
	CTRL	EB	EBY	2X
Item, %DM				
Corn silage	45.0	45.0	45.0	45.0
Bermuda grass hay	7.00	7.00	7.00	7.00
Corn grain	27.0	27.0	27.0	27.0
Soybean meal	20.5	20.5	20.5	20.5
Mineral Mix	0.50	0.50	0.50	0.50
Additive, mg/d				
Bacteria^*b*^	.	0.74	0.74	1.484
Enzymes^*c*^	.	0.95	0.95	1.908
Yeast^*d*^	.	.	48.07	96.142
Total	.	1.70	49.77	99.534
Chemical composition, %DM				
CP	16.4	16.4	16.4	16.4
NDF	28.3	28.3	28.3	28.3
ADF	15.2	15.2	15.2	15.2
Starch	30.5	30.5	30.5	30.5
EE	2.20	2.20	2.20	2.20

^*a*^Treatments: CTRL = no additives; EB = addition of enzymes and bacteria; EBY = addition of enzymes, bacteria, and yeast; 2X = addition of enzymes, bacteria, and yeast at double the EBY dosage.

^*b*^Bacteria strains included in the pack were *Lactobacillus animalis*, *Propionibacterium freudenreichii*, *Bacillus lichenformis*, *Bacillus subtilis*, and *Enterococcus faecium*.

^*c*^Enzyme pack included 0.58 mg hemicellulose, 0.21 mg xylanase, and 0.16 mg amylase.

^*d*^Yeast contained 10.49 mg live yeast and 37.58 mg yeast culture both derived from *Saccharomyces cerevisiae*.

For preparation of the experimental diets, the corn silage was dried for 72 h at 60 °C in a forced-air oven (Heratherm, Thermo Scientific, Waltham, MA) and all ingredients were ground to 2 mm particle size in a Wiley mill (model N°2; Arthur H. Thomas Co., Philadelphia, PA). One sample of each feed was further ground to 1 mm particle size for chemical analyses. The doses of additives (Table 1) were defined based on manufacturer guidelines and are comparable to the ones used in other studies (Leicester et al., 2016; Oeztuerk et al., 2005; Oh et al., 2019) reflecting practical feeding protocols currently used by dairy nutritionists in the United States. The bacterial culture/enzyme blend contained five strains of bacteria with a combined 1 × 10^9^.

CFU (*Lactobacillus animalis*, *Propionibacterium freudenreichii*, *Bacillus lichenformis*, *Bacillus subtilis*, and *Enterococcus faecium*) and three enzymes (amylase [27,837 U amylose], hemicellulase [55.33 U xylose; 638.8 U mannose], and xylanase [58,598 U]). Enzyme activity is expressed in U, and one U defined as 1 μmol of substrate released per minute. The enzymes were derived from *Aspergillus oryzae* and *Trichoderma reesei*. The yeast component contained a mixture of live and culture yeast of the TS20 strain *Saccharomyces cerevisiae* with a CFU of 4.0 × 1010. Treatments were fed at the following doses: EB at 1.7 mg, EBY at 49.76 mg, and 2X at 99.53 mg/d. The doses were selected according to manufacturer guidelines and are comparable to the ones used in other studies (Leicester et al., 2016; Oeztuerk et al., 2005; Oh et al., 2019) as well as reflecting practical feeding protocols currently used by dairy nutritionists in the United States.

### Dual-flow Continuous Culture System Operation

For this experiment we used a dual-flow continuous culture based on the system originally developed by Hoover et al. (1976) and recently used for ruminal microbiome studies (Arce-Cordero et al., 2022a; Monteiro et al., 2022). Conditions were maintained at continuous agitation (100 rpm), infusion of N_2_ gas to displace oxygen, constant temperature (39 °C), and infusion of artificial saliva (Weller and Pilgrim, 1974) with 0.40 g/L of urea, at 3.05 mL per minute to individually regulate passage rates of liquid (11% h^−1^) and solid (5.5% h^−1^) effluents of digesta.

This experiment consisted of a total of 40 d of fermentation divided in 4 fermentation periods of 10 d each. Fermenters were inoculated on day one of each fermentation period with ruminal contents collected from two cannulated Holstein cows in mid-lactation that were fed a total mixed ration twice per day containing 38% corn silage, 19% ground corn, 13% soybean meal, 11% cottonseed, 9% citrus pulp, 8.5% mineral premix, and 1.5% palmitic acid supplement. Ruminal contents were manually collected from each cow 2 h after the morning feeding, strained through 2 layers of cheesecloth, transferred into pre-warmed thermos jars, and immediately transported to the lab. Each fermenter was pre-warmed and under continuous flush of N_2_ gas at the moment of inoculation with approximately 1.82 L of a 50:50 mix (v/v) of ruminal contents from both cows.

Each fermenter was provided 106 g DM d^−1^ of the corresponding experimental diet, distributed equally into two portions of 53 g DM at 0800 and 1800 h. The yeast products and hemicellulase were added as dry products to their respective diets and divided into two equal doses. The bacteria culture and remaining enzymes were added to distilled water solutions to ensure accurate dosing due to the small amounts needed in the diet. Fresh solutions were prepared at 0700 h every day and were pipetted into the fermenters immediately before both morning and evening feedings.

### Collection of Data and Samples

The first 7 d of fermentation of each period were used for adaptation to experimental diets and stabilization of bacterial communities (Salfer et al., 2018). Collection of data and samples was performed on d 8, 9, and 10 of each period. On those same days, containers of solid and liquid digesta effluent were kept in an ice-cold water bath and digesta temperature maintained at −2 °C to preserve the quality of the samples.

Samples for bacterial sequencing analysis were collected separately from liquid and solid effluents of each fermenter every day at 3, 6, and 9 h after morning feed provision. For the liquid fraction, a total of 45 mL per fermenter per day were collected (15 mL at each timepoint). For the solid fraction, 200 g of solid effluent were collected at each timepoint and strained through 4 layers of cheesecloth, totaling an approximate of 25 g of solid sample collected from each fermenter per day. All samples were stored at −80 °C for subsequent DNA extraction.

Samples were also collected for VFA analyses at the end of each day of fermentation. Liquid and solid effluents of each fermenter were combined and strained through 4 layers of cheesecloth. A 10 mL sample of strained fluid was acidified with 100 µL of 50% H_2_SO_4_ and stored at −20 °C for subsequent analyses of propionate and butyrate.

### Laboratory Analyses

#### Chemical composition of feed ingredients.

 Samples of feed ingredients for experimental diets were analyzed for: DM (AOAC, 1990; method 930.15), total N (AOAC, 2000; method 990.03) by rapid combustion with a micro elemental N analyzer (Vario Micro Cube, Elementar, Hanau, Germany), NDF (Van Soest et al., 1991) adapted for Ankom^200^ Fiber Analyzer (Ankom Technology, Macedon, NY) with heat-stable α-amylase and sodium sulphite, total starch by enzymatic hydrolysis (Hall, 2015), and ether extract (AOAC, 2000; method 2003.05) determined by a fat analyzer (XT20, Ankom Technology).

#### Concentration of butyrate and propionate.

 Molar proportions of propionate and butyrate used as an input for the correlation analysis of the current study were calculated as follows: (individual VFA m*M*/ total VFA m*M*) × 100 and reported in our companion study Bennett et al. (2021). Briefly, samples for VFA analyses were processed according to Ruiz-Moreno et al. (2015) by centrifuging at 10,000 × *g* for 15 min. Supernatant was mixed with a solution of crotonic acid and metaphosphoric acid to freeze overnight, and then centrifuged again at 10,000 × *g* for 15 min. Resulting supernatant was mixed with ethyl acetate, vortexed and the top layer transferred to a chromatography injection vial for gas chromatography (Agilent 7820A GC, Agilent Technologies, Palo Alto, CA) with a flame ionization detector and a capillary column (CP-WAX 58 FFAP 25 m 0.53 mm, Varian CP7767, Varian Analytical Instruments, Walnut Creek, CA) maintained at 110 °C, with injector temperature at 200 °C and detector at 220 °C.

#### DNA extraction.

 Samples were thawed at room temperature and combined across days and timepoints within the same period and fermenter, resulting in 64 samples total (32 samples of liquid effluent fraction and 32 samples of solid effluent fraction). Genomic DNA of liquid and solid effluent samples was extracted separately following the methodology by Stevenson and Weimer (2007) and described by Arce-Cordero et al. (2022a) for samples of continuous culture fermenters. For each solid sample, 22 g were blended with extraction buffer (Tris HCl, ethylenediaminetetraacetic acid, and NaCl) and centrifuged at 500 × *g* for 15 min at 4 °C. Resulting supernatant from solid samples was processed following the same protocol used for liquid samples by centrifuging 22 mL at 10,000 × *g* for 25 min at 4 °C and resuspending the bacterial pellet in DNA extraction buffer.

Bacterial pellets were mixed with 20% sodium lauryl sulfate solution and phenol and processed by repeated bead beating (Biospec Products) using zirconium beads (BioSpec Products, Bartlesville, OK). The DNA was extracted through sequential centrifugations with phenol, phenol/chloroform, and chloroform; and precipitated with 3 *M* Na acetate buffer and isopropanol. After centrifugation with 70% ethanol, the DNA pellet was resuspended in Tris-EDTA buffer. Concentration of DNA samples was measured with Qubit Fluorometer (Invitrogen, San Diego, CA) and stored at −80 °C.

#### DNA amplification and sequencing.

 The V4 hypervariable region of bacterial 16S rRNA gene was amplified using dual-index primers (Caporaso et al., 2011) according to Kozich et al. (2013). The PCR amplification reaction consisted of 1 µL forward index primer (10 m*M*), 1 µL reverse index primer (10 m*M*), 1 µL DNA template (10 ng/µL), and 17 µL Pfx AccuPrime master mix (Invitrogen, USA). The reaction protocol consisted of denaturation for 5 min at 95 °C, followed by 30 cycles of 95 °C for 30 s, annealing at 55 °C for 30 s, extension at 72 °C for 1 min, and elongation for 5 min at 72 °C. The amplicons were run on a 1% agarose gel to confirm success of the PCR and normalized with a SequalPrep Normalization Plate Kit (Applied Biosystems, Foster City, CA) to construct the DNA pool library. A total of 64 samples were sequenced at the Interdisciplinary Center for Biotechnology Research (ICBR) of the University of Florida using a MiSeq reagent kit V2 (2 × 250 cycles run; Illumina, San Diego, CA, USA) in an Illumina MiSeq platform (Illumina, San Diego, CA, USA). Sequencing data were deposited into the NCBI database with the following accession number PRJNA854650.

### Bacterial Sequence Data Analysis

Data were analyzed with Quantitative Insights into Microbial Ecology version 2 (QIIME 2) pipeline (Bolyen et al., 2019). Paired-end raw reads were imported and quality of the initial bases was evaluated with the Interactive Quality Plot. The divisive Amplicon Denoising Algorithm (DADA2) pipeline implemented in QIIME 2, was used for sequence quality control including steps for filtering low quality reads, denoising reads, merging paired-end reads, and removing chimeric reads. The phylogenetic tree was generated with align-to-tree-mafft-fasttree pipeline from the q2-phylogeny plugin of QIIME 2. Sequencing depth was normalized to 10,800 sequences per sample and the number of amplicon sequence variants (ASVs), richness (Chao1), diversity (Shannon index), and Bray-Curtis distance were calculated by the core-metrics-phylogenetic method. Resulting ASVs were classified into phylum, class, order, family, and genus, using the q2-feature-classifier plugin of QIIME 2 and the SILVA 138 database (https://www.arb-silva.de/documentation/release-1381/). Only average relative abundances greater than 0.1% were considered for analyses.

### Statistical Analysis

The results of bacterial community structure were visualized with principal component analysis plots (PCoA) based on comparisons using Bray-Curtis distance and analyzed with R vegan package (Callahan et al., 2016). The effects of treatments in community structure and alpha diversity were determined with the PERMANOVA test implemented in QIIME 2. Log-transformed data of taxa relative abundance were analyzed with the MIXED procedure of SAS 9.4 (SAS Institute Inc., Cary, NC). The statistical model included the fixed effect of treatment and random effects of period, square, and fermenter. Orthogonal contrasts were used to test the effects of 1) ADD—the control compared to all treatments with additives (CTRL vs. EB, EBY, and 2X); 2) YEAST—treatment without yeast compared to those with yeast (EB vs. EBY and 2X); and 3) DOSE—the single dose of enzymes, bacteria, and yeast compared to the doubled dose (EBY vs. 2X). Correlations between molar proportions of propionate and butyrate in ruminal fluid and relative abundance of genera affected by addition of additives were analyzed using the Pearson correlation procedure. Significance was declared at *P* ≤ 0.05, while 0.05 < *P* ≤ 0.10 was considered a trend.

## RESULTS AND DISCUSSION

Overall, in this study 64 samples were sequenced consisting of 32 samples of liquid fraction and 32 samples of solid fraction. A total of 1,558,835 reads were generated from 16S rRNA sequencing, out of which 1,232,414 high-quality sequences were retained for analysis after filtering, denoising, merging, and removing chimeras with DADA2 pipeline. In the solid fraction a total of 19 phyla, 34 classes, 64 orders, 97 families, and 219 genera were identified. For the liquid fraction 20 phyla, 35 classes, 70 orders, 110 families, and 229 genera were identified across samples.

**Figure 1.**
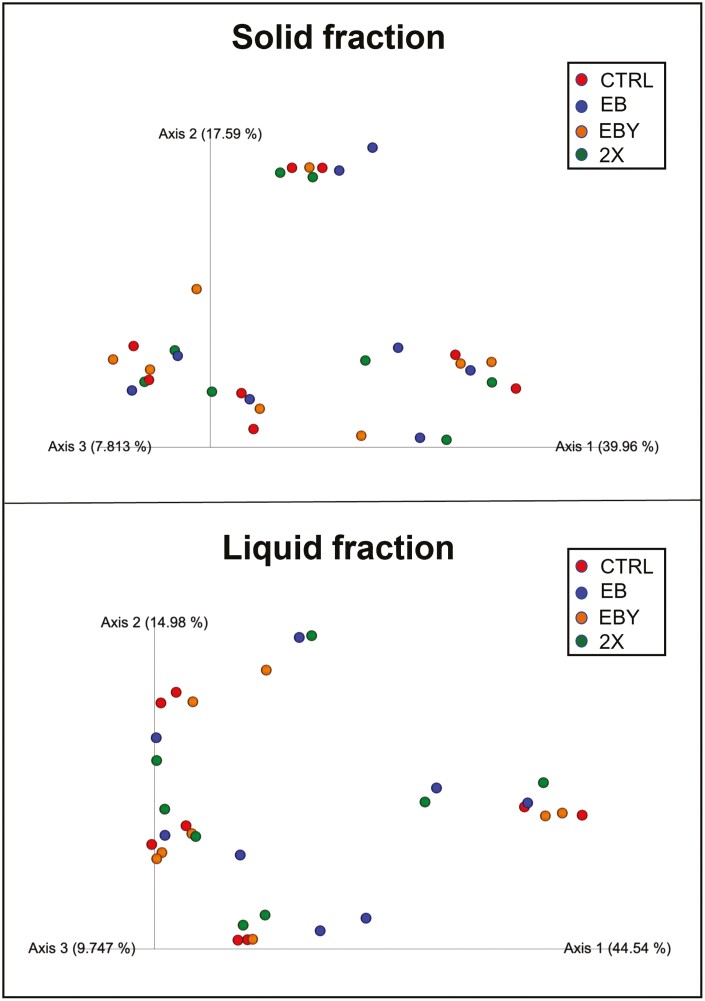
Principal coordinates analysis (PCoA) plots of Bray-Curtis similarity comparing the treatment effects on community structure of ruminal bacteria. Treatments: CTRL = no additives; EB = addition of enzymes and bacteria; EBY = addition of enzymes, bacteria, and yeast; 2X = addition of enzymes, bacteria, and yeast at double the EBY dosage.

**Figure 2.**
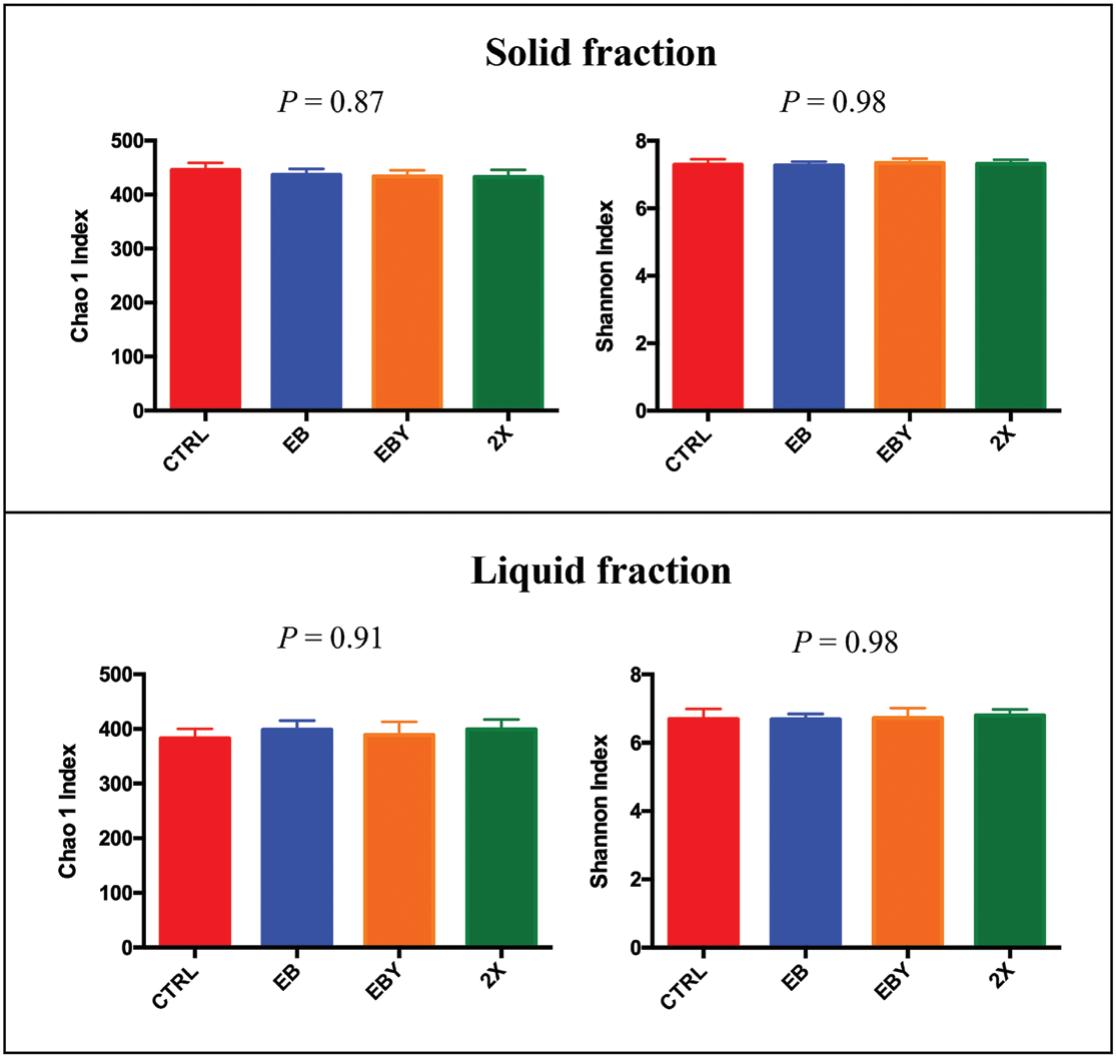
Effects of experimental treatments on alpha diversity of ruminal bacteria. Treatments: CTRL = no additives; EB = addition of enzymes and bacteria; EBY = addition of enzymes, bacteria, and yeast; 2X = addition of enzymes, bacteria, and yeast at double the EBY dosage.

The effect of treatments on bacterial community structure of solid and liquid fractions is presented in Figure 1. Based on Bray-Curtis similarity index, there were no effects of treatments on bacterial community structure of neither of the fractions analyzed. Similarly, we did not observe a treatment effect on alpha diversity of neither solid nor liquid fractions (Figure 2), indicating that microbial populations of samples analyzed presented similar richness and diversity regardless of incorporation of additives in the diet. Given the lack of larger effects of treatments on microbial community structure and alpha and beta diversities, we decided to evaluate the effects of microbial additives and enzymes on microbial relative abundance at the phylum, family, and genera levels.

At the phylum level (Table 2) in the solid fraction *Firmicutes, Bacteroidota, Proteobacteria, Spirochaetota*, and *Actinobacteriota*, were the five most abundant phyla which accounted on average for 50.8, 23.3, 10.1, 8.6, and 2.2% of the sequences, respectively. Moreover, relative abundance of phyla in the liquid fraction was also dominated by *Firmicutes, Bacteroidota, Proteobacteria, Spirochaetota, and Actinobacteriota*, which accounted for relative abundances of 40.7, 29.3, 20.2, 3.7, and 3.0%, respectively.

**Table 2.** Effects of bacterial cultures, enzymes, and yeast additives on relative abundance of main phyla of bacteria in solid and liquid fractions

Phylum	Treatment means^*a*^				SEM	*P*-value^*b*^		
	CTRL	EB	EBY	2X		ADD	YEAST	DOSE
Solid fraction								
*Firmicutes*	52.4	49.6	50.3	50.8	1.32	0.09	0.49	0.70
*Bacteroidota*	22.2	23.2	24.4	23.3	1.33	0.14	0.53	0.36
*Proteobacteria*	10.3	11.09	9.73	9.39	2.41	0.79	0.12	0.76
*Spirochaetota*	8.63	8.55	8.86	8.3	1.38	0.92	0.96	0.39
*Actinobacteriota*	1.46	2.6	1.55	3.25	1.23	0.26	0.83	0.12
Liquid fraction								
*Firmicutes*	41.5	39.2	41.9	40.2	2.40	0.28	0.11	0.16
*Bacteroidota*	28.7	30.2	29.0	29.5	3.06	0.56	0.55	0.77
*Proteobacteria*	21.1	20.5	19.6	19.5	5.13	0.49	0.60	0.97
*Spirochaetota*	4.0	3.3	4.3	3.0	1.43	0.37	0.45	0.06
*Actinobacteriota*	1.5	3.9	2.2	4.4	1.92	0.18	0.72	0.22

^*a*^Treatments: CTRL = no additives; EB = addition of enzymes and bacteria; EBY = addition of enzymes, bacteria, and yeast; 2X = addition of enzymes, bacteria, and yeast at double the EBY dosage.

^*b*^Orthogonal contrasts: ADD = CTRL vs. EB, EBY and 2X; YEAST = EB vs. EBY and 2X; and DOSE = EBY vs. 2X.

In the liquid fraction, we did not observe any effects of microbial additives and enzymes on phylum relative abundance. However, addition of microbial additives and enzymes (ADD) tended to decrease the relative abundance of *Firmicutes* in the solid fraction (Table 2). Lower relative abundance of *Firmicutes* was reported by Monteiro et al. (2022) for bacteria in the liquid fraction as a result of adding either *Lactobacillus plantarum* or a blend of *Lactobacillus acidophilus* and *Propionibacterium freudenreichii* to corn silage-based diets in continuous culture. Conversely, greater relative abundance of *Firmicutes* in ruminal fluid has been reported by Plaizier et al. (2017) in cows with subacute ruminal acidosis resulting from a reduction of dietary NDF concentration, suggesting a positive association between *Firmicutes* relative abundance and ruminal acidity. However, Arce-Cordero et al. (2022a) found the opposite observing that a reduction in dietary NDF from 40% to 30% promoted a lower relative abundance of *Firmicutes* in the solid fraction. The fact that phylum is such as large taxonomic category including a wide diversity of bacteria, makes it difficult to associate changes in relative abundance at the phylum level with specific ruminal or dietary conditions. Therefore, we analyzed the effects of microbial additives and enzymes of our experiment at smaller taxonomic levels, such as family and genus.

The effects of microbial additives and exogenous enzymes on the most abundant families of bacteria are presented in Table 3. Relative abundance of families in the solid fraction was dominated by *Lachnospiraceae* (26.6%), *Prevotellaceae* (16.9%), *Succinivibrionaceae* (10.2%), *Spirochaetaceae* (8.66%), and *Ruminococcaceae* (5.15). Moreover, in the liquid fraction we found that *Prevotellaceae* (20.9%), *Succinivibrionaceae* (20.0%), *Lachnospiraceae* (17.8%), *Selenomonaceae* (4.1%), and *Spirochaetaceae* (3.60%), were the most abundant families. Previous studies have already reported *Prevotellaceae*, *Lachnospiraceae*, *Succinivibrionaceae*, and *Spirochaetaceae* within the most abundant families in both solid and liquid fractions of ruminal cultures (Arce-Cordero et al., 2022a; Monteiro et al., 2022) and ruminal samples (Söllinger et al., 2018).

**Table 3.** Effects of bacterial cultures, enzymes, and yeast additives on relative abundance of main families of bacteria in solid and liquid fractions

Family	Treatment means^*a*^				SEM	*P*-value^*b*^		
	CTRL	EB	EBY	2X		ADD	YEAST	DOSE
Solid fraction								
*Lachnospiraceae*	26.6	26.1	26.4	25.8	1.49	0.62	0.97	0.62
*Prevotellaceae*	16.9	18.9	19.5	18.6	0.82	0.04	0.87	0.46
*Succinivibrionaceae*	10.2	10.9	9.55	9.26	2.43	0.76	0.12	0.79
*Spirochaetaceae*	8.66	8.66	8.90	8.29	1.34	0.93	0.90	0.36
*Ruminococcaceae*	5.15	4.18	3.91	4.93	0.85	0.13	0.66	0.12
*Acidaminococcaceae*	4.60	4.04	4.59	3.84	0.67	0.21	0.64	0.09
*Selenomonadaceae*	3.36	3.70	3.89	3.66	0.41	0.32	0.85	0.63
*Christensenellaceae*	2.61	2.50	2.55	2.55	0.28	0.74	0.85	1.00
*Fibrobacteraceae*	1.98	2.04	2.26	2.14	0.31	0.62	0.66	0.77
*Oscillospiraceae*	1.96	1.73	1.85	1.85	0.36	0.40	0.52	1.00
Liquid fraction								
*Prevotellaceae*	20.4	21.8	20.9	20.8	1.32	0.55	0.45	0.94
*Succinivibrionaceae*	20.9	20.3	19.5	19.4	5.14	0.51	0.63	0.94
*Lachnospiraceae*	17.5	17.3	17.9	18.4	0.99	0.63	0.33	0.59
*Selenomonadaceae*	4.24	4.11	4.55	3.61	1.31	0.80	0.96	0.20
*Spirochaetaceae*	3.98	3.29	4.30	3.01	1.41	0.41	0.51	0.06
*Rikenellaceae*	4.01	3.16	3.59	3.74	1.34	0.17	0.21	0.74
*Ruminococcaceae*	3.43	2.78	3.11	3.43	1.00	0.63	0.49	0.70
*Oscillospiraceae*	3.54	2.75	3.31	3.10	0.67	0.08	0.11	0.51
*Acidaminococcaceae*	2.99	2.75	3.06	2.94	0.48	0.78	0.35	0.68
*Bifidobacteriaceae*	1.11	3.29	1.69	3.70	1.57	0.16	0.65	0.19

^*a*^Treatments: CTRL = no additives; EB = addition of enzymes and bacteria; EBY = addition of enzymes, bacteria, and yeast; 2X = addition of enzymes, bacteria, and yeast at double the EBY dosage.

^*b*^Orthogonal contrasts: ADD = CTRL vs. EB, EBY and 2X; YEAST = EB vs. EBY and 2X; and DOSE = EBY vs. 2X.

For the solid fraction, we found that the combination of microbial additives and enzymes (ADD) increased the relative abundance of *Prevotellaceae*. Ruminal bacteria of *Prevotellaceae* family have been primarily associated with propionate synthesis. Deusch et al. (2017) found that microbial proteins associated with propionate production were dominated by enzymes from *Bacteroidetes* species, mainly from the *Prevotellaceae* family. Conversely, results from our previous study (Bennett et al., 2021) showed that the combination of microbial additives and exogenous enzymes (ADD) tended to decrease propionate molar proportion and increased butyrate molar proportion. Although synthesis of propionate is a well-known role of bacteria of *Prevotellaceae* family, it is also known that these bacteria, particularly some strains of *Prevotella*, produce xylanase which allows them to utilize xylan and pectins very efficiently (Stewart et al., 1997), which may favor an increase in molar proportion of butyrate.

Moreover, *Prevotellacea* was found to be the most abundant family in the liquid fraction and the second most abundant after *Lachnospiraceae* in the solid fraction of our study. Previous research has already reported *Prevotellacea* as one of the most abundant families of bacteria in the rumen (Deusch et al., 2017; Stevenson and Weimer, 2007). Considering that *Firmicutes* and *Bacteroidota* are the two main phyla in the rumen, and that family *Prevotellacea* belongs to phylum *Bacteroidota*, one could speculate that the lower relative abundance of *Firmicutes* observed in the solid fraction of our study, as a result of the addition of microbial additives and exogenous enzymes to the diet (ADD), may be at least in part explained by the increase in relative abundance of *Prevotellaceae*.

Results of the effects of bacterial cultures, enzymes, and yeast additives on relative abundance of genera in the solid fraction are presented in Table 4. Addition of microbial additives and exogenous enzymes to the diet (ADD), decreased the relative abundance of *Acetitomaculum* and *Saccharofermentans*, and conversely increased the relative abundance of *Prevotellaceae Ga6A1*, *Prevotellaceae YAB2003*, *Anaerovibrio*, *Mogibacterium*, and *Bifidobacterium*. Decreased relative abundance of bacteria associated with synthesis of acetate and cellulose degradation, such as *Acetitomaculum* (Le Van et al., 1998) and *Saccharofermentans* (Mu et al., 2021) may indicate a stimulatory effect of microbial additives and enzymes (ADD) on bacterial taxa associated with either degradation of non-structural carbohydrates or synthesis of VFA other than acetate. The simultaneous increase in relative abundances observed for *Prevotellaceae Ga6A1*and *Prevotellaceae YAB2003* (both increases of approximately 0.8 percentage units) clearly contribute to the 2.1 percentage units increase in relative abundance observed for *Prevotellaceae* family in the solid fraction (Table 3).

**Table 4.** Effects of bacterial cultures, enzymes, and yeast additives on relative abundance of main genera of bacteria in solid fraction

Genus	Family	Treatment means^*a*^				SEM	*P*-value^*b*^		
		CTRL	EB	EBY	2X		ADD	YEAST	DOSE
*UCG-001*	*Succinivibrionaceae*	6.36	8.40	6.41	5.94	3.04	0.60	0.06	0.72
*Acetitomaculum*	*Lachnospiraceae*	5.56	3.93	4.34	4.29	1.44	0.03	0.53	0.94
*Succiniclasticum*	*Acidaminococcaceae*	4.51	4.04	4.54	3.78	0.66	0.25	0.74	0.07
*Ga6A1_group*	*Prevotellaceae*	1.90	2.76	2.89	2.51	0.36	<0.01	0.81	0.22
*YAB2003_group*	*Prevotellaceae*	1.65	2.41	2.33	2.69	0.35	<0.01	0.70	0.21
*Ruminococcus*	*Ruminococcaceae*	2.34	1.98	2.23	2.25	0.31	0.18	0.08	0.88
*Saccharofermentans*	*Hungateiclostridiaceae*	1.88	1.43	1.66	1.61	0.23	0.03	0.14	0.76
*CAG-352*	*Ruminococcaceae*	1.74	1.33	0.84	1.68	0.95	0.16	0.84	0.04
*Pseudoscardovia*	*Bifidobacteriaceae*	0.84	1.39	0.64	2.34	1.00	0.39	0.89	0.06
*UCG-003*	*Prevotellaceae*	0.71	0.68	0.85	0.80	0.09	0.40	0.06	0.58
*Anaerovibrio*	*Selenomonadaceae*	0.41	0.48	0.56	0.55	0.09	0.09	0.26	0.88
*Mogibacterium*	*Anaerovoracaceae*	0.38	0.44	0.50	0.48	0.04	0.02	0.23	0.60
*Bifidobacterium*	*Bifidobacteriaceae*	0.14	0.70	0.41	0.30	0.20	0.08	0.09	0.62

^*a*^Treatments: CTRL = no additives; EB = addition of enzymes and bacteria; EBY = addition of enzymes, bacteria, and yeast; 2X = addition of enzymes, bacteria, and yeast at double the EBY dosage.

^*b*^Orthogonal contrasts: ADD = CTRL vs. EB, EBY and 2X; YEAST = EB vs. EBY and 2X; and DOSE = EBY vs. 2X.

Multiple roles have been found for ruminal bacteria within the *Prevotellaceae* family, ranging from degradation of polysaccharides and protein to utilization of monosaccharides, accounting for most of the enzymes involved in acetate and propionate synthesis in the rumen (Deusch et al., 2017). Recently, Bach et al. (2019) found a sharp increase in relative abundance of some genera within the *Prevotellaceae* family (including *YAB2003*) in the rumen of dairy cows when transitioned from a dry cow diet into a lactation diet at the time of calving. However, the nutritional versatility that enables bacteria within the *Prevotellaceae* family to thrive over a wide range of substrates and ruminal conditions, may impose a challenge for interpretation of the roles of genera and species within this taxonomic group (Stevenson and Weimer, 2007).

As an approach to evaluate how complex microbial ecosystems respond to changes, some studies have evaluated quorum sensing (QS), which is a mechanism of communication among gram negative bacteria, where acyl homoserine lactone (AHL)-based QS seems to be an important signaling system which has been found in the ruminal environment (Erickson et al., 2002). In this regard, Won et al. (2020) showed that *Butyrivibrio*, *Prevotella*, *Ruminococcus*, and *Pseudobutyrivibrio*, which are the most abundant bacterial genera in the rumen, have the capacity to use (AHL)-based QS. Moreover, they found that *Prevotella* in particular plays a very important role in QS within the rumen based on the expression levels of LuxS synthase gene (regulator of AHL signal), highlighting the importance of *Prevotella* for the adaptation of ruminal microbiome to different conditions, which may explain the results of our study where relative abundance of members of the *Prevotellaceae* family is positively correlated with butyrate molar proportion, differing from their role in propionate synthesis that has been reported in other studies.

Lower relative abundance of *Succinivibrionaceae UCG-001* and greater abundance of *Ruminococcus* and *Prevotellaceae UCG-003* were observed in the solid fraction as a result of yeast supplementation (Table 4). Previous research demonstrates that abundance of members of *Succinivibrionaceae* family and their metaproteome increases in the rumen of cows as a result of greater intake of grain (Deusch et al., 2017), which is consistent with Anderson (1995) who reported that some members of *Succinivibrionaceae* family as strictly starch degrading microorganisms without affinity for glucose or nonstarch polysaccharides. On the other hand, *Ruminococcus* is one of the most important cellulose degraders in the rumen (La Reau and Suen, 2018), whose abundance is greater in animals consuming diets with a greater proportion of forage (Henderson et al., 2015; Zeng et al., 2019). These results are consistent with Desnoyers et al. (2009) meta-analysis reporting that the effect of yeast supplementation on organic matter digestibility in dairy cows increases with proportion of NDF in the diet, suggesting an important role of cellulose degrading microorganisms on the response of dairy cows to yeast.

Results of the relative abundance of genera in the liquid fraction as affected by addition of bacterial cultures, enzymes, and yeast additives to the diet are summarized in Table 5. In consistency with the results observed for the solid fraction, in the liquid fraction we also found greater relative abundance of *Prevotellaceae Ga6A1* and lower abundance of *Saccharofermentans* as a result of adding microbial additives and enzymes to the diet (ADD). Additionally, we observed a greater relative abundance of *Unclassified Lachnospiracea* and *Ruminococcus gauvreauii*, along with lower abundance of *Rikenellaceae RC9*, *NK4A214*, and *Bacteroidales BS11* in response to microbial additives and enzymes (ADD). According to La Reau and Suen (2018), *Ruminococcus gauvreauii*, was originally classified as a member of family *Ruminococcus*; however, current molecular techniques have revealed that it actually belongs to the *Lachnospiraceae* family, therefore, our data shows that addition of microbial additives and enzymes (ADD) increased two genera of the *Lachnospiraceae* family which has been associated with ruminal synthesis of butyrate (Cotta and Forster, 2006; Meehan and Beiko, 2014) and is consistent with the findings of our previous study (Bennett et al., 2021).

**Table 5.** Effects of bacterial cultures, enzymes, and yeast additives on relative abundance of main genera of bacteria in liquid fraction

Genus	Family	Treatment means^*a*^				SEM	*P*-value^*b*^		
		CTRL	EB	EBY	2X		ADD	YEAST	DOSE
*Treponema*	*Spirochaetaceae*	3.96	3.13	4.23	3.00	1.40	0.33	0.39	0.07
*RC9_gut_group*	*Rikenellaceae*	3.75	2.90	3.39	3.30	1.31	0.09	0.20	0.82
*Lachnobacterium*	*Lachnospiraceae*	1.76	1.51	1.59	2.36	0.74	0.87	0.23	0.09
*NK4A214_group*	Oscillospiraceae	1.98	1.46	1.66	1.55	0.46	0.02	0.42	0.58
*Unclassified*	*Lachnospiraceae*	1.14	1.61	2.36	1.35	0.55	0.09	0.53	0.03
*Pseudoscardovia*	*Bifidobacteriaceae*	0.76	1.78	0.68	3.03	1.24	0.27	0.94	0.05
*Unclassified*	*Selenomonadaceae*	0.75	0.75	1.06	0.48	0.56	0.96	0.94	0.05
*Family_XIII_AD3011_group*	*Anaerovoracaceae*	0.69	0.49	0.69	0.64	0.13	0.09	0.00	0.40
*Ga6A1_group*	*Prevotellaceae*	0.34	0.50	0.58	0.45	0.17	0.08	0.90	0.28
*Saccharofermentans*	*Hungateiclostridiaceae*	0.50	0.39	0.38	0.46	0.07	0.10	0.58	0.19
*Ruminococcus_gauvreauii_group*	*Lachnospiraceae*	0.24	0.48	0.44	0.40	0.17	0.10	0.66	0.80
*Unclassified*	*Bacteroidales_BS11_gut_group*	0.50	0.30	0.30	0.18	0.16	0.04	0.60	0.37
*FD2005*	*Lachnospiraceae*	0.35	0.38	0.16	0.24	0.15	0.29	0.06	0.47

^*a*^Treatments: CTRL = no additives; EB = addition of enzymes and bacteria; EBY = addition of enzymes, bacteria, and yeast; 2X = addition of enzymes, bacteria, and yeast at double the EBY dosage.

^*b*^Orthogonal contrasts: ADD = CTRL vs. EB, EBY and 2X; YEAST = EB vs. EBY and 2X; and DOSE = EBY vs. 2X.

**Figure 3.**
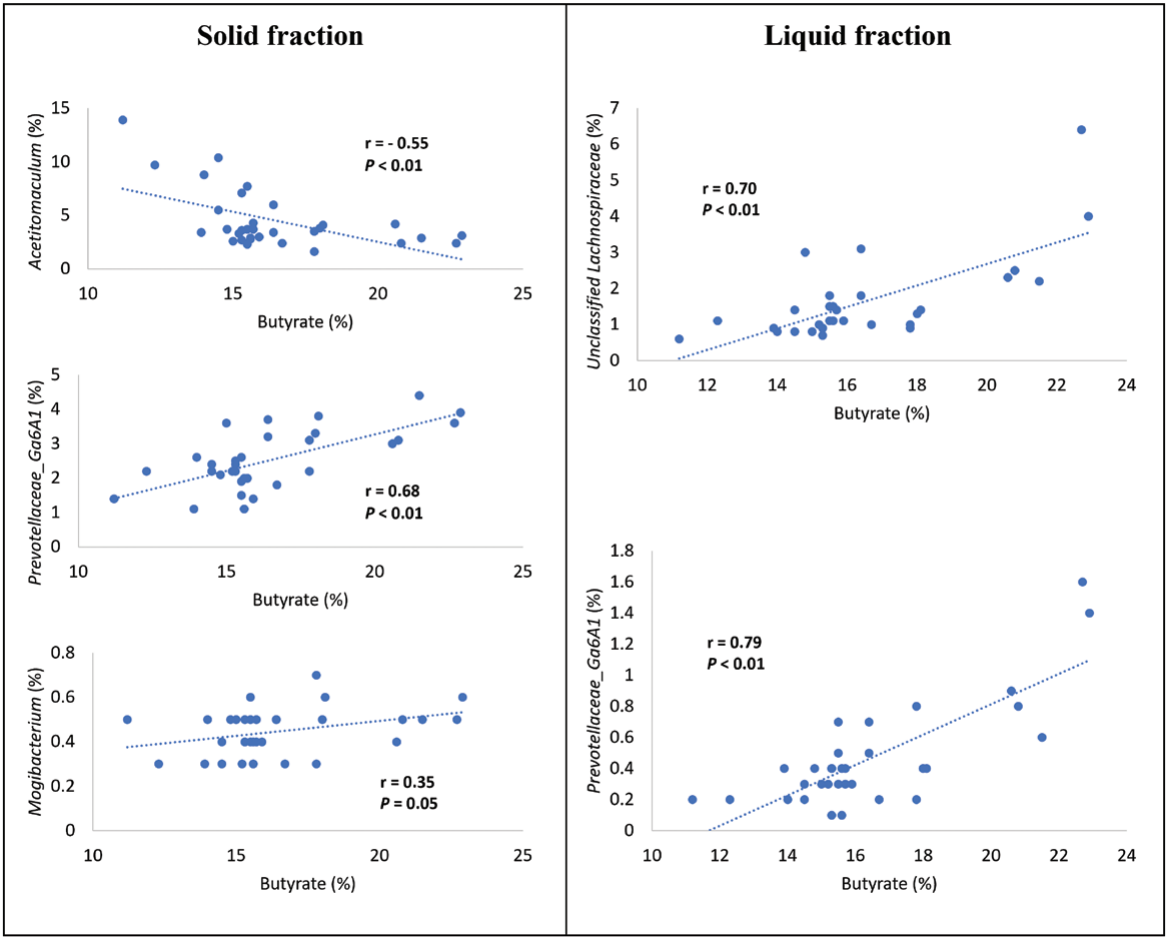
Correlation between molar proportion of butyrate in ruminal fluid and relative abundance of genera in liquid and solid fraction. Molar proportion of butyrate is expressed as: (butyrate m*M*/ total VFA m*M*) × 100.

Figure 3 summarizes data on the correlation between molar proportion of butyrate in ruminal fluid and relative abundance of genera in the solid and liquid fractions. In the solid fraction, relative abundance of acetate producer *Acetitomaculum* was negatively correlated (*r* = −0.55) with molar proportion of butyrate, while relative abundances of *Prevotellaceae Ga6A1* and *Mogibacterium* exhibited a positive correlation (*r* = 0.68 and *r* = 0.35, respectively). It has been reported that reductive acetogenesis performed by *Acetitomaculum* competes for H_2_ against methanogenic archaea (Le Van et al., 1998), indicating that ruminal conditions that promote low methanogenesis and greater synthesis of propionate may stimulate growth of *Acetitomaculum*. Consistently, in our previous study we found that combination of microbial additives and enzymes (ADD) tended to decrease the molar proportion of propionate and increase butyrate synthesis (Bennett et al., 2021), which may have favored a greater abundance of *Acetitomaculum* and its indirect negative correlation with butyrate molar proportion.

Relative abundance of *Prevotellaceae Ga6A1* was highly correlated with molar proportion of butyrate in both solid and liquid fractions (Figure 3; *r* = 0.68 and *r* = 0.79, respectively). In agreement with the increase in relative abundance of *Prevotellaceae Ga6A1* observed in both fractions (Tables 4 and 5) and the greater molar proportion of butyrate in our previous study (Bennett et al., 2021) resulting from addition of microbial additives and enzymes to the diet (ADD), these data suggest that some members of family *Prevotellacea* may also play a role on ruminal synthesis of butyrate. Similarly, in a recent study evaluating alkalizing effects of magnesium sources Arce-Cordero et al. (2022b) reported that *Prevotellaceae Ga6A1* tended to be less abundant in the treatment that resulted in the lowest molar proportion of butyrate. Moreover, for the liquid fraction, a positive correlation between butyrate molar proportion and *Unclassified Lachnospiraceae* was found (*r* = 0.70), which may also be an important driver in the responses observed in our study considering the role of *Lachnospiraceae* family members in butyrate synthesis (Cotta and Forster, 2006).

To a lesser extent in comparison to the combined effect of microbial additives and enzymes (ADD), some effects of addition of yeast (YEAST), and increase of dose (DOSE) were also observed at the genus level. Addition of yeast to the diet (YEAST) decreased the relative abundance of *Succinivibrionaceae UCG-001* in the solid fraction and *Lachnospiraceae FD2005* in the liquid fraction; while it increased the relative abundance of *Ruminococcus* and *Prevotellaceae UCG-003* in the solid fraction and *Anaerovoracaceae Family XIII AD301* in the liquid fraction. Members of *Succinivibrionaceae* family, have been associated to ruminal synthesis of succinate that can be further metabolized into propionate through the succinate decarboxylation pathway (Paynter and Elsden, 1970; Ungerfeld, 2020). A positive correlation between molar proportion of propionate with *Succinivibrio* and *Lachnospiraceae FD2005* has been reported previously for dual flow experiments (Arce-Cordero et al., 2022a). Although we did not observe an effect of YEAST on propionate (Bennett et al., 2021), which is consistent with meta-analyses (Desnoyers et al., 2009; Oh et al., 2019) that report similar propionate concentration and greater ruminal pH with yeast supplementation, our results indicate that *Succinivibrionaceae UCG-001* may be playing an important role for the effects of yeasts on ruminal fermentation, especially considering the high relative abundance of *Succinivibrionaceae UCG-001*, which averaged 6.8% across the samples of our experiment.

Increasing the dose of exogenous enzymes and microbial additives (DOSE) decreased the relative abundance of *Succiniclasticum* in the solid fraction and *Treponema* and *Selenomonadaceae* in the liquid fraction. Conversely, the relative abundance of *Ruminococcaceae CAG-352*, *Oscillospirales*, and *Pseudoscardovia* in the solid fraction, and *Lachnobacterium*, *Pseudoscardovia* in the liquid fraction, was greater when the dose increased. Both *Succiniclasticum* and *Selenomonadaceae* have been shown to actively metabolize succinate into propionate in the rumen (Gylswyk, 1995). Although we did not observe an effect of DOSE in molar proportion of propionate (Bennett et al., 2021) there may have been some contribution of the 2X treatment to the overall effect of enzymes and microbial additives (ADD) on reducing propionate molar proportion. Moreover, *Ruminococcaceae CAG-352* and *Lachnobacterium* have been identified as cellulolytic (Deusch et al., 2017) and lactate producing (Ricci et al., 2022) bacteria, respectively; which challenges the explanation of a clear effect of DOSE on microbiome.

In conclusion, our results demonstrate that the addition of yeast (YEAST) decreases relative abundance of bacteria involved in synthesis of succinate, while doubling the dose of enzymes, bacteria and yeast (DOSE) decreases the abundance of bacteria that metabolize succinate into propionate. Moreover, the combination of exogenous enzymes, bacteria, and yeast as additives to corn silage-based diets (ADD) has an effect on ruminal microbiome, primarily by increasing the relative abundance of specific genera of bacteria within the *Prevotellaceae* family, which may explain the increase in butyrate molar proportion observed with ADD. Overall, this research contributes to the current understanding of the animal response to supplementation of exogenous enzymes and microbial additives.
